# Caveolin-1 promotes Ewing sarcoma metastasis regulating MMP-9 expression through MAPK/ERK pathway

**DOI:** 10.18632/oncotarget.10872

**Published:** 2016-07-28

**Authors:** Laura Lagares-Tena, Silvia García-Monclús, Roser López-Alemany, Olga Almacellas-Rabaiget, Juan Huertas-Martínez, Miguel Sáinz-Jaspeado, Silvia Mateo-Lozano, Carlos Rodríguez-Galindo, Santiago Rello-Varona, David Herrero-Martín, Oscar M. Tirado

**Affiliations:** ^1^ Sarcoma Research Group, Institut d'Investigació Biomèdica de Bellvitge-IDIBELL, L'Hospitalet de Llobregat, Barcelona, Spain; ^2^ Developmental Tumor Biology Laboratory, Hospital Sant Joan de Deu, Barcelona, Spain; ^3^ Pediatric Hematology/Oncology, Dana-Farber/Boston Children's Cancer and Blood Disorders Center, Harvard Medical School, Boston, Massachusetts, USA

**Keywords:** caveolin-1, Ewing sarcoma, metastasis, mmp9, mapk

## Abstract

Ewing sarcoma (ES) is a bone and soft tissue sarcoma affecting mostly children and young adults. Caveolin-1 (CAV1) is a well-known target of EWS/FLI1, the main driver of ES, with an oncogenic role in ES. We have previously described how CAV1 is able to induce metastasis in ES via matrix metalloproteinase-9 (MMP-9). In the present study we showed how CAV1 silencing in ES reduced MEK1/2 and ERK1/2 phosphorylation. Accordingly, chemical inhibition of MEK1/2 resulted in reduction in MMP-9 expression and activity that correlated with reduced migration and invasion. IQ Motif Containing GTPase Activating Protein 1 (IQGAP1) silencing reduced MEK1/2 and ERK1/2 phosphorylation and *MMP-9* expression. Furthermore, IQGAP1 silenced cells showed a marked decrease in their migratory and invasive capacity. We demonstrated that CAV1 and IQGAP1 localize in close proximity at the cellular edge, thus IQGAP1 could be the connecting node between CAV1 and MEK/ERK in ES metastatic phenotype. Analysis of the phosphorylation profile of CAV1-silenced cells showed a decrease of p-ribosomal protein S6 (RPS6). RPS6 can be phosphorylated by p90 ribosomal S6 kinases (RSK) proteins. CAV1-silenced cells showed reduced levels of p-RSK1 and treatment with U0126 provoked the same effect. Despite not affecting ERK1/2 and RPS6 phosphorylation status neither *MMP-9* expression nor activity, RSK1 silencing resulted in a reduced migratory and invasive capacity *in vitro* and reduced incidence of metastases *in vivo* in a novel orthotopic model. The present work provides new insights into CAV1-driven metastatic process in ES unveiling novel key nodes.

## INTRODUCTION

ES is the second most common bone tumor in children and adolescents. This tumor is very aggressive and highly metastatic. Approximately, one third of ES patients present metastasis at diagnosis, being lung and bone marrow the most common sites. The treatment and prognosis of ES patients are determined among other factors by the presence of metastases. The 5-year survival rate of metastatic patients ranges from 20 to 45% depending on location (lung and bone/bone marrow respectively), compared to 60-70% of those with localized disease [1]. Therefore, in order to find new therapeutic targets, further advances in the knowledge of ES metastatic key regulators are mandatory.

ES tumors have a distinctive chromosomal translocation that gives rise to a fusion protein, most commonly EWS/FLI1 (85% of cases) [2, 3]. These fusion proteins act as aberrant transcription factors, deregulating the expression of several target genes, therefore affecting different pathways involved in the initiation, maintenance and progression of the tumor [4–6]. Our group has previously described CAV1 as one of these target genes, demonstrating its role in the malignant phenotype of ES [7], promoting cell proliferation [8], angiogenesis [9], metastasis [10] and chemotherapy resistance [11].

Matrix metalloproteinases (MMPs) are implicated in extracellular matrix remodeling and have been related to metastasis progression [12–13]. Especially MMP-9 has been related to tumor aggressiveness in different tumor types, including sarcomas [14]. In ES CAV1 seems to regulate indirectly MMP-9 transcription [10], thus promoting tumor metastasis. MEK/ERK is a pathway constitutively activated in ES [15], thus ERK1/2 could be a putative mediator of CAV1 pro-invasive role. Hence, the goal of this study is to further understand the mechanism through which CAV1 exerts its role in ES metastatic behavior.

## RESULTS

### MEK1/2 and ERK1/2 phosphorylation is reduced in CAV1-knockdown cells

**Figure 1:**
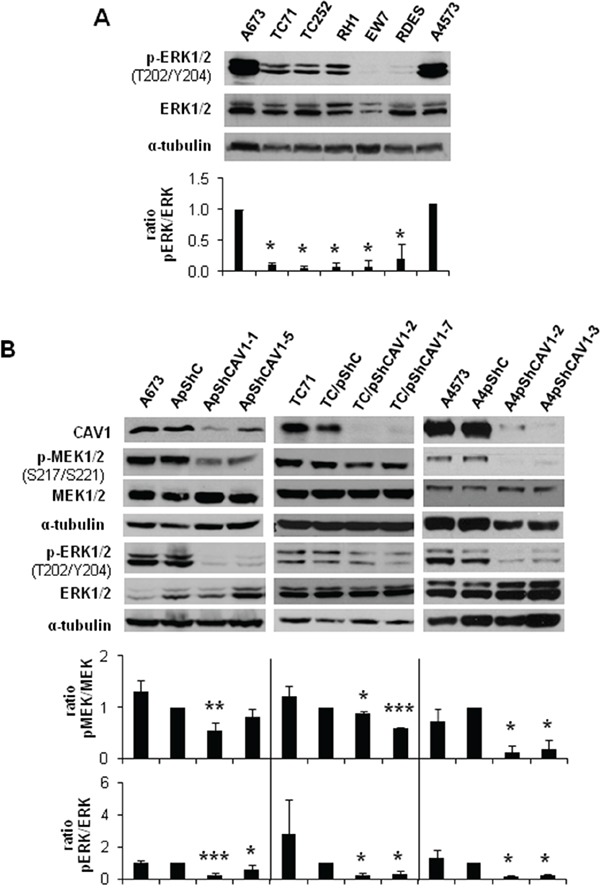
MEK1/2 and ERK1/2 phosphorylation is reduced in CAV1-knockdown cells **A.** Representative western blot showing the phosphorylation and expression levels of ERK1/2 in different ES cell lines. **B.** Representative blots of MEK1/2 and ERK1/2, either total or phosphorylated, in A673, TC71 and A4573 CAV1-silenced models. α-tubulin was used as loading control. Quantification of the phosphorylated *vs*. total ratios is shown below each set of blots. Statistical significance was achieved by the Student's *t* test: *p≤0.05 **p≤0.001 ***p≤0.0001.

The MEK/ERK pathway has been previously related with *MMP-9* expression and regulation of invasion [16]. As A673 ES cells bear an activating mutation (V599E) in *B-Raf*, ERK1/2 could be a good candidate for MMP-9 regulation [17]. As expected, we detected in A673 cells more phosphorylated ERK1/2 than in other ES cell lines harbouring the same EWS/FLI1 fusion type (TC71, TC252, RH1, EW7) (Figure 1A). Moreover, we found a decrease in ERK1/2 phosphorylation in our previously published CAV1-knockdown models (Figure 1B). We also observed a reduction in the phosphorylated form of the direct activator of ERK1/2, MEK1/2 (Figure 1B). These results correlated with an inhibition of MMP-9 at the transcriptional level (Supplementary Figure S1) [10].

**Figure 2:**
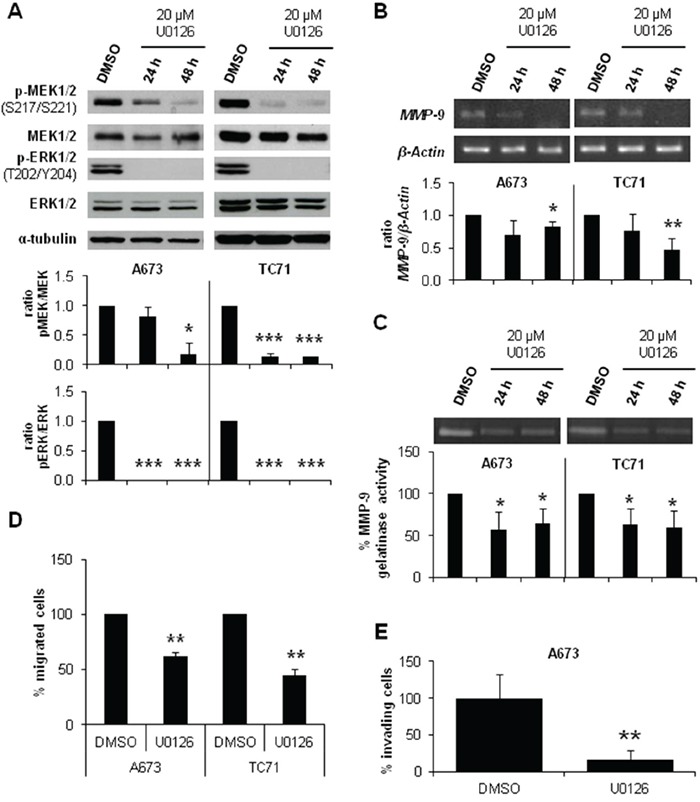
MEK1/2 inhibitor U0126 reduced *MMP-9* expression and activity and diminished migration and invasion in ES cell lines **A.** Representative blots of MEK1/2 and ERK1/2, either total or phosphorylated, in A673 and TC71 cell lines treated with vehicle (DMSO) or 20 μM U0126. Quantification of the phosphorylated *vs*. total ratios is shown below the blot. **B.** Semi-quantitative RT-PCR and corresponding quantifications showing *MMP-9/β-Actin* ratio in A673 and TC71 cell lines treated with vehicle (DMSO) or 20 μM U0126. **C.** Zymogram and corresponding quantification of the MMP-9 gelatinase activity in A673 and TC71 cell lines treated with vehicle (DMSO) or 20 μM U0126. **D.** Quantification of the transwell migratory capacity of A673 and TC71 treated cells (U0126) regarding their controls (DMSO). **E.** Quantification of the matrigel-coated transwell invasive capacity of A673 and TC71 treated cells (U0126) regarding their controls (DMSO). Statistical significance was achieved by the Student's *t* test: *p≤0.05 **p≤0.001 ***p≤0.0001.

We further investigated the involvement of MEK/ERK pathway in the reduced invasive phenotype described in CAV1-knockdown ES models [10]. We treated A673 and TC71 cells with a specific MEK1/2 inhibitor, U0126. As expected, after 24 h we observed a decrease in MEK1/2 and ERK1/2 phosphorylation levels as shown in Figure 2A. The same inhibitory effect was confirmed in *MMP-9* expression and proteolytic activity (Figure 2B and 2C). Additionally, U0126-treated cells migrated significantly less than the untreated ones (Figure 2D) and showed a lower invasive capacity (Figure 2E), thus partially reproducing the observed phenotype in CAV1-silenced cells. These observations strongly suggest that p-ERK1/2 could be implicated in the CAV1-dependent regulation of MMP-9 transcription in ES cells.

### IQGAP1 links CAV1 and MEK/ERK in ES migration and invasion

**Figure 3:**
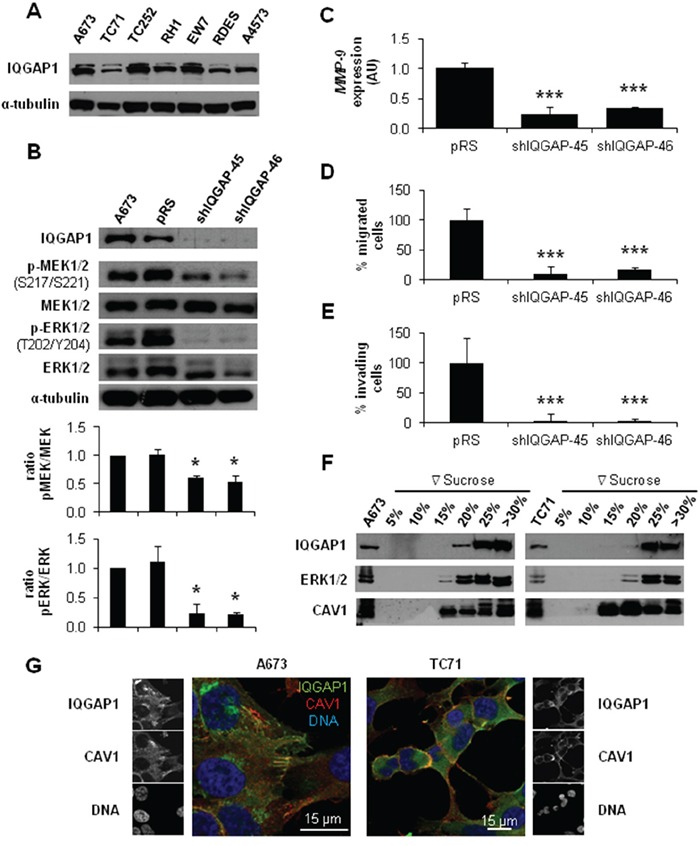
IQGAP1 intermediates in CAV1-regulation of migratory and invasive capacity of ES cells **A.** Representative western blot showing the expression levels of IQGAP1 in different ES cell lines. **B.** Representative blots of MEK1/2 and ERK1/2, either total or phosphorylated, in IQGAP1-interfered cells. α-tubulin was used as loading control in every panel. Quantification of the phosphorylated *vs*. total ratios is shown below each set of blots **C.** Quantitative real time RT-PCR showing *MMP-9* levels (*β-Actin* as reference) in A673 shIQGAP1 model. **D.** Quantification of the transwell migratory capacity in A673 IQGAP1-interfered cells. **E.** Quantification of the matrigel-coated transwell invasive capacity in A673 IQGAP1-silenced model. **F.** Western blot of IQGAP1, ERK1/2 and CAV1 in sucrose gradient separation phases from A673 (left) and TC71 (right) cell lines. **G.** Confocal imaging showing IQGAP1 (green) and CAV1 (red) in A673 (left) and TC71 (right) cell lines. Scale bar 15 μm. Statistical significance was achieved by the Student's *t* test: *p≤0.05 **p≤0.001 ***p≤0.0001.

IQGAP1 has been involved in invasion [18] and linked to ERK2 modulating its activity [19]. Thus, IQGAP1 could be playing a similar role in ES. First, we checked that all ES cell lines expressed IQGAP1 (Figure 3A). Then, we obtained a stable silencing model of IQGAP1 in the ES cell line A673. IQGAP1-silenced cells showed a reduction in p-MEK1/2 and p-ERK1/2 levels (Figure 3B). Likewise, knockdown of IQGAP1 diminished *MMP-9* expression and the migratory and invasive capacity of A673 cells (Figure 3C-3E). Finally, by sucrose gradient and co-immunofluorescence (IF) experiments in A673 and TC71 cells we were able to show a close pattern of distribution of IQGAP1 and CAV1 at the cellular edge, particularly in membrane protusions (Figure 3F and 3G). These results suggest that IQGAP1 could be acting as a scaffold between CAV1 and MEK/ERK pathway in the metastatic phenotype of ES cells.

### RPS6 and RSK1 phosphorylation is decreased in CAV1-silenced cells

**Figure 4:**
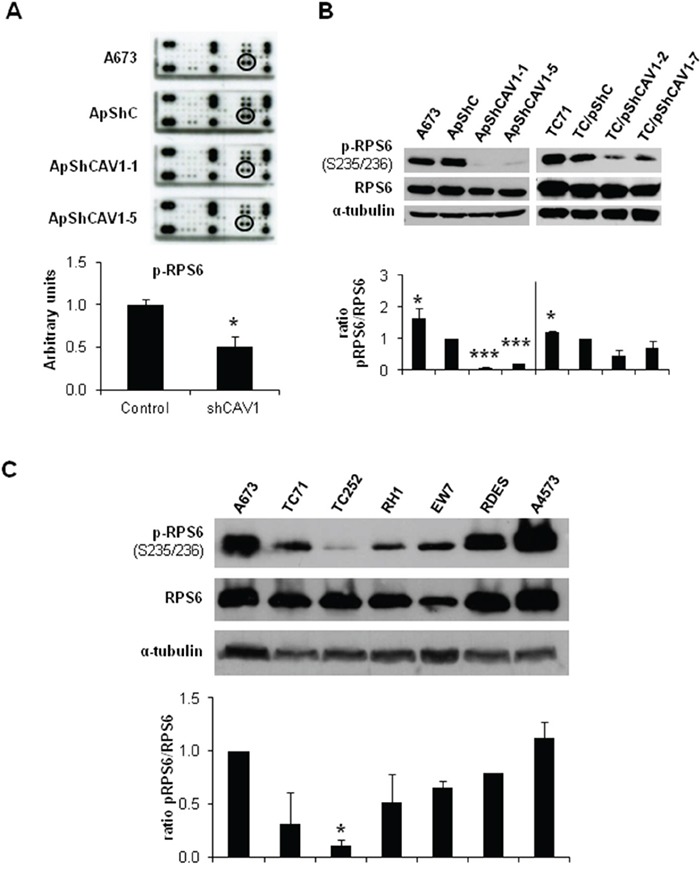
RPS6 phosphorylation is decreased in CAV1-silenced cells **A.** RTK Signaling Antibody Array performed with A673 CAV1-knockdown model. Quantification data of p-RPS6 expression corresponds to the circled spots. **B.** Representative blots of RPS6, either total or phosphorylated, in shCAV1 models. α-tubulin was used as loading control. **C.** Representative western blot showing the phosphorylation and expression levels of RPS6 in different ES cell lines. α-tubulin was used as loading control. Quantification of the phosphorylated *vs*. total ratios is shown below each set of blots. Statistical significance was achieved by the Student's *t* test: *p≤0.05 **p≤0.001 ***p≤0.0001.

**Figure 5:**
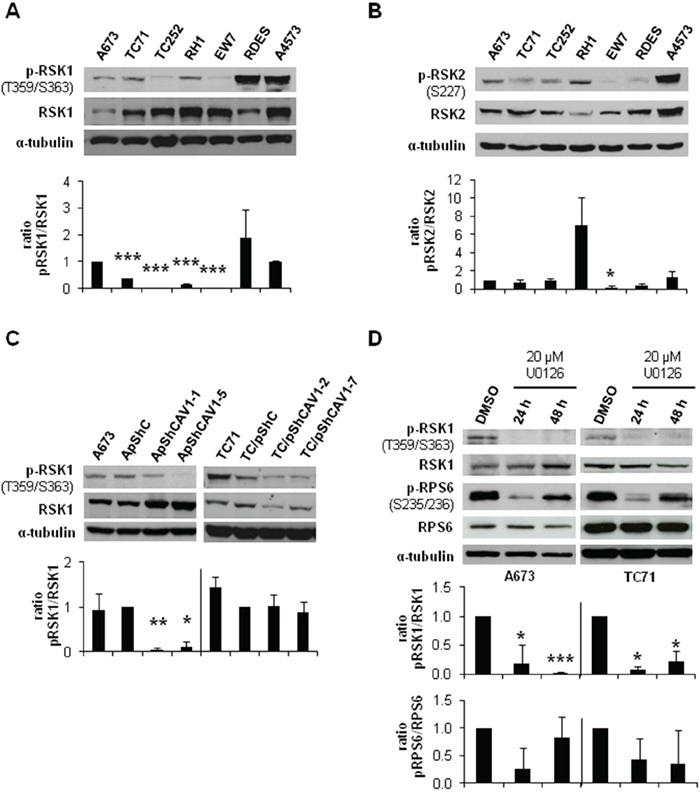
CAV1-knockdown and MEK1/2 inhibition reduced RSK1 phosphorylation levels **A.** Representative western blot showing the phosphorylation and expression levels of RSK1 in different ES cell lines. **B.** Representative western blot showing the phosphorylation and expression levels of RSK2 in different ES cell lines. **C.** Representative blots of RSK1, either total or phosphorylated, in shCAV1 models. **D.** Representative blots of RSK1 and RPS6, either total or phosphorylated, in A673 and TC71 cell lines treated with vehicle (DMSO) or 20 μM U0126. α-tubulin was used as loading control in every panel. Quantification of the phosphorylated *vs*. total ratios is shown below each set of blots. Statistical significance was achieved by the Student's *t* test: *p≤0.05 **p≤0.001 ***p≤0.0001.

The activation of ERK1/2 pathway can be triggered by different membrane receptors [20]. We analyzed the phosphorylation profile of the A673 CAV1-knockdown model focusing on several Tyr-kinase receptors, as well as different intracellular signaling nodes. Surprisingly, the only significant change that we were able to detect was a reduction in RPS6 phosphorylation (Figure 4A) (Supplementary Table S1). The decrease in p-RPS6 was then confirmed in two CAV1-silenced models (Figure 4B). Both RPS6 and p-RPS6 are well expressed in ES cell lines (Figure 4C). We proceeded to check, by immunohistochemistry (IHC), the status of p-RPS6 in a tissue microarray (TMA) of 26 ES patients [21–22]. We found a significant correlation between high p-RPS6 levels and prolonged survival of ES patients (Supplementary Figure S2, Supplementary Table S2). Although RPS6 is described as a main target of the mTOR pathway it can also be phosphorylated on Ser235/236 by RSK proteins in an ERK1/2-dependent manner [23]. RSK family proteins are involved in several cellular functions such as cell cycle regulation, migration and survival [24–25]. RSK family comprises four members, RSK1-4, that can be phosphorylated by ERK1/2 [24]. ES cell lines express similar levels of RSK1 although there are higher variations regarding RSK2 (Figure 5A and 5B); however, neither RSK3 nor RSK4 were detected (data not shown). We could appreciate clear differences in the phosphorylation status of RSK1 and RSK2 among the ES cell lines tested (Figure 5A and 5B). We focused our attention in the expression and phosphorylation status of RSK1 in A673 and TC71 CAV1-silenced models. RSK1 phosphorylation levels were reduced in shCAV1 cells (Figure 5C). Furthermore, when treated during 24 h with U0126, not only RSK1 but also RPS6 phosphorylation levels were reduced in both cell lines (Figure 5D).

### RSK1 silencing did not affect RPS6 phosphorylation nor *MMP-9* expression and activity

**Figure 6:**
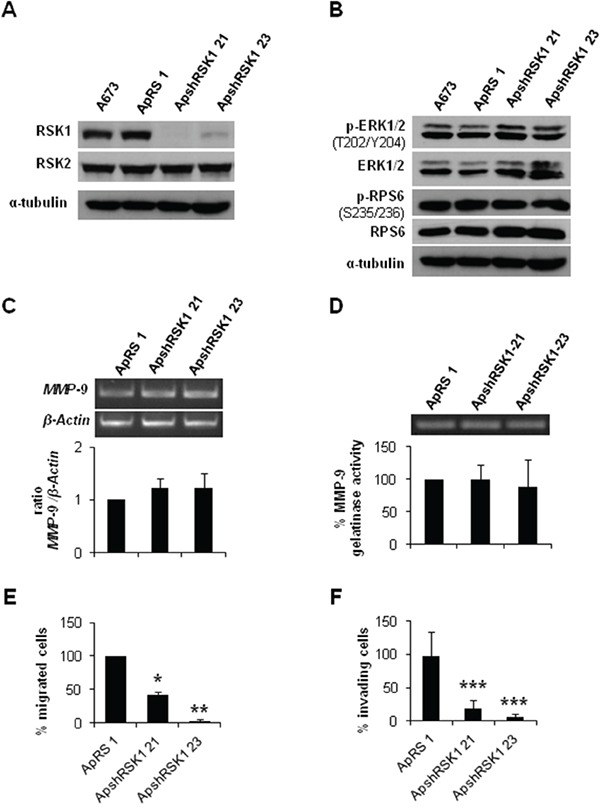
RSK1 silencing did not affect *MMP-9* expression but abrogate both migration and invasion of ES cells **A.** Immunoblot of RSK1 expression levels in A673 shRSK1. **B.** Representative blots of RSK1 and RPS6, either total or phosphorylated, in RSK1-silenced cells. α-tubulin was used as loading control. **C.** Semi-quantitative RT-PCR and corresponding quantification showing *MMP-9/β-Actin* ratio in A673 RSK1-knockdown model. **D.** Zymogram and corresponding quantification of the MMP-9 gelatinase activity in A673 shRSK1 cells. **E.** Quantification of the transwell migratory capacity of A673 RSK1-interfered cells. **F.** Quantification of the matrigel-coated transwell invasive capacity of A673 RSK1-knockdown low expression model. Statistical significance was achieved by the Student's *t* test: *p≤0.05 **p≤0.001 ***p≤0.0001.

As we observed lower RSK1 phosphorylation in CAV1-knockdown cells, we analyzed the specific role of this protein in the CAV1-depentent *MMP-9* regulation in ES cells. With that purpose, we established a specific and stable RSK1-knockdown model in the A673 cell line. RSK1 silencing was effectively achieved without affecting RSK2 levels (Figure 6A). RSK1 interference did not cause any variation in the phosphorylation levels of ERK1/2 and RPS6 (Figure 6B) neither in *MMP-9* expression and/or activity (Figure 6C and 6D). Nonetheless, RSK1-knockdown seemed to affect the migratory and the invasive capacity of shRSK1 A673 cells *in vitro* as both were drastically diminished (Figure 6E and 6F). Although RPS6 phosphorylation and *MMP-9* regulation in ES seems to be independent of RSK1 these results confirmed the important role of RSK1 in ES migration and invasion.

### RSK1 silencing reduced the incidence of metastases *in vivo*

**Figure 7:**
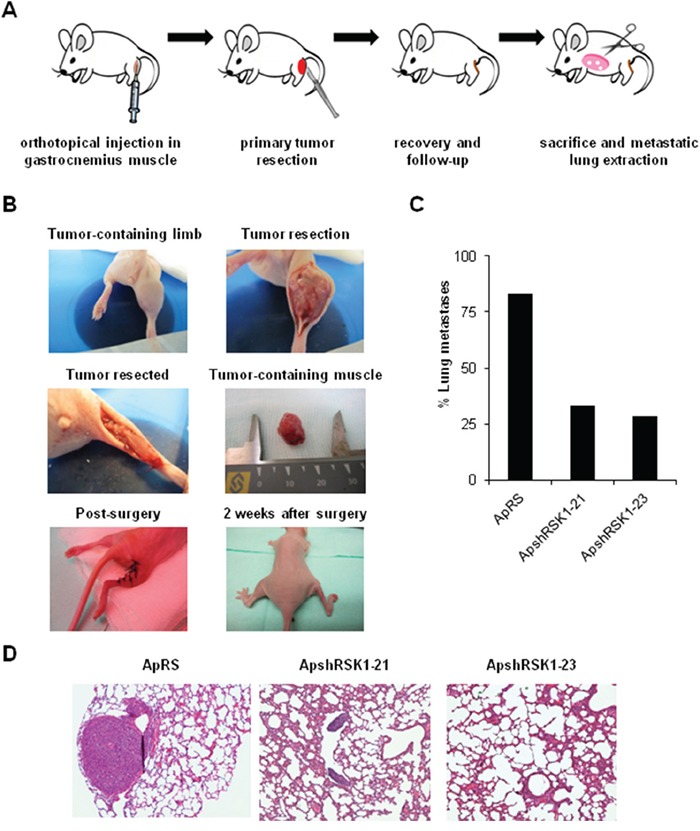
RSK1 silencing reduced the incidence of metastases *in vivo* in an ES orthotopical xenograft model **A** and **B.** Scheme and pictures depicting A673 RSK1-knockdown model after orthotopical implantation inside gastrocnemius muscle and removal of muscle after primary tumor growth. **C.** Lung metastasis incidence in A673 RSK1-silenced cells after primary tumor removal. **D.** Representative images of lungs recovered from mice orthotopically injected with A673 shRSK1 cells (magnification, ×100). Drawings in Figure 7A are adapted from graphics deposited in Wikimedia Commons and Servier Medical Art under a Creative Commons Attribution 3.0 Unported License.

Our *in vitro* results suggest that RSK1 might have effects on migration and invasion of ES cells. Both steps are crucial for the occurrence of metastasis. Therefore, we sought whether RSK1 silencing has any effect *in vivo* using an experimental metastatic assay. This first attempt based on i.v. injection of A673 RSK1-knockdown cells failed to show any difference on lung colonization (Supplementary Figure S3). Thus, we developed a modified version of a previously described orthotopic model [26], as we consider that this new methodological approach recapitulates more accurately the steps of metastasis in ES patients. Cells were injected into the gastrocnemius muscles of BALB/c^*nu/nu*^. Once primary tumor-bearing limbs reached a volume of 800 mm^3^, gastrocnemius muscles were surgically resected to reduce morbidity associated with excessive tumor growth and to allow metastases to form. A summary of the model and images of each step are depicted in Figure 7A and 7B. For all the different types of cells injected (shRSK1 *vs*. control), the frequency of primary tumor occurrence was 100%, thus showing no differences regarding growth rate or tumor size. Tumors were extracted between 18-20 days after inoculation and mice were sacrificed 60 days post-injection, lungs extracted and the number of metastases counted. After histological examination of the lungs, 83% of mice injected with control cells showed presence of metastases, while in RSK1-silenced clones the metastases incidence was reduced to 33.33 % and 28.57% (Figure 7C and 7D). These *in vivo* results are in concordance with those obtained *in vitro*, as RSK1 silencing has a drastic effect on migration and invasion and point to RSK1 as a key pro-metastatic agent in ES.

## DISCUSSION

We have previously reported the oncogenic and pro-invasive role of CAV1 in ES and how *MMP-9* expression is reduced once CAV1 is silenced [7, 10]. MEK/ERK pathway is involved in several cellular processes as cell adhesion, cell cycle regulation, migration, survival, differentiation, proliferation, metabolism and/or protein expression [27]. *MMP-9* regulation through ERK1/2 has been well characterized in a variety of cellular models [16, 28–29]. ERK1/2 is activated and has been related to metastasis in several cancers as hepatocellular carcinoma [30], lung cancer [31] or colorectal carcinoma [32]. Besides, constitutive activation of this pathway has been described in ES [15, 33]. ERK1/2 activity is regulated by CAV1 in different tumor types. For example CAV1 silencing results in a decrease in ERK1/2 phosphorylation in colon cancer [34] and in metastatic lung cancer cells [35].

In the present work we have verified the activation of ERK1/2 in most of ES cell lines and how the phosphorylated forms of ERK1/2 and its precursor MEK1/2 were reduced in CAV1-silenced ES cells. Moreover, when treated with MEK1/2 inhibitor U0126, A673 and TC71 cells showed, besides p-MEK1/2 and p-ERK1/2 inhibition, lower *MMP-9* expression, reduced proteolytic activity and cellular migration and invasion were impeded. These results are the same previously described in the CAV1-knockdown models [10]; thus we have found a correlation in ES between MEK1/2 and ERK1/2 phosphorylation inhibition and a reduction in *MMP-9* expression and proteolytic activity. ERK1/2 has been linked with important targets of EWS/FLI-1 as IGF-1 [36] or tumor suppressor LOX [37]. Indeed, U0126 increased sensitivity to IGF-1R antibody figitumumab while MEK/ERK activation via p-ERK1/2 (Thr202/Tyr204) contributes to IGF1R-related therapies resistance in metastatic ES [38–39]. All these facts give evidence of the crucial role of MEK/ERK pathway in the pro-metastatic effect that CAV1 exerts through MMP-9 in ES cells.

IQGAP1 has been described as a scaffold for MAPK signaling pathway [40]. Scaffold proteins coordinate many intracellular signaling processes assuring the correct flow of information inside the cell [41]. IQGAP1 behaves as a modulator of several cellular mechanisms as cytoskeleton organization or cellular adhesion, interacts with ERK2 and MEK1/2 [19, 40] and has been associated with proliferation [42] and invasion [43]. First, we verified the expression of IQGAP1 in the ES cell lines. Stable silencing of IQGAP1 in A673 cells resulted in a reduction in p-MEK1/2 and p-ERK1/2 levels besides a marked decrease of *MMP-9* expression, migration and invasion. We showed that IQGAP1 and CAV1 share localization at membrane protusions in ES cells. These results suggest that IQGAP1 could be the hub connecting CAV1 and MEK/ERK in ES as it has been already described in other neoplasias [44].

Analysis of the phosphorylation profile of A673 shCAV1 model revealed a reduction in RPS6 phosphorylation as the only significant change among Tyr-kinase receptors. Lower p-RPS6 levels were also detected in TC71 shCAV1 cells. RPS6 is a cytoplasmic 40S ribosomal protein that belongs to the S6E family of ribosomal proteins. RPS6 is a major substrate in the ribosome of protein kinases and it is involved in cell growth and proliferation via translation of specific mRNAs [23]. In six of the seven ES cell lines checked RPS6 was phosphorylated. In a set of ES patients we observed a positive correlation between high p-RPS6 levels and improved survival. Our observation is in agreement with what it has been previously described in other ES patients set: the association between p-mTOR protein overexpression and better survival [39].

RPS6 can be phosphorylated on Ser235/236 by RSK proteins in an ERK1/2-dependent manner [23]. The four RSK isoforms have been implicated in several neoplasias [24]. Although ES cell lines show almost the same level of RSK1 there are evident differences within them concerning the phosphorylation forms of RSK1 and RSK2. CAV1 silencing in A673 and TC71 cells diminished p-RSK1 as U0126 treatment did. A673 and TC71 cells treatment with U0126 during 24 h caused also a decrease in phospho-RPS6 levels but were recovered 24 h later. We could not detect any p-RPS6 variation in A673 RSK1-knockdown cells regarding controls, thus in ES RPS6 phosphorylation seems to be RSK1-independent. RSK1-knockdown did not change p-ERK1/2 levels and neither affected *MMP-9* expression or activity. Therefore, RSK1 seems not to be implicated in *MMP-9* regulation in ES. Nevertheless, RSK1 is affecting the migratory and invasive capacity of A673 cells. Our results agree with previous studies that show the crucial role of RSK1 in the migratory phenotype of epithelial [45] or melanoma cells, promoting migration through RhoA inhibition [46]. Conversely, in lung cancer cells RSK1 silencing increases cell motility [47]. So, RSK1 may have contrary effects on migration capacity depending on the cellular background, being proactive in ES cells. RSK1 inhibition did not affect p-ERK1/2 and p-RPS6 status and neither *MMP-9* expression nor its activity. Moreover, the experimental metastasis model showed no differences between RSK1 silenced cells and controls, suggesting that RSK1 has no role on the ability of lung colonization of ES cells. However, we could verify in a new orthotopic model that the metastatic capacity of A673 shRSK1 cells is not only reduced *in vitro* but also *in vivo*. Therefore, our results point to RSK1 as an important pro-metastatic player in ES as it has been previously described in other tumors [48–49]. We are also presenting for the first time this modified version of a previously described orthotopic model [26]. We believe that this orthotopic model recapitulates more closely the metastatic steps in ES as the primary tumor grows in its natural environment. Resection of the gastrocnemius is a low-aggressive surgery that allows the survival of the mice with a normal mouse life for a period long enough for the development of distant metastases. Thus, this animal model may become a valuable experimental tool to analyze metastatic potential not only in ES but in other sarcomas too.

In summary, we demonstrate that CAV1 regulates the expression and activity of *MMP-9* through the MEK/ERK pathway in ES being IQGAP1 the putative connecting node. We have also unveiled RPS6 as an important signaling node under CAV1 influence. Moreover we showed that RSK1 is a key player in ES metastasis. A full comprehensive understanding of ES metastatic process is mandatory to develop novel therapeutic strategies. Thus, as several clinically tested MEK/ERK inhibitors are available, ES patients could benefit from their application.

## MATERIALS AND METHODS

### Cell culture and stable transfections

A673, TC252 (gifts from Dr. Heinrich Kovar, Children's Cancer Research Institute, Vienna, Austria), RH1 (gift from Dr. Peter Houghton, Greehey Children's Cancer Research Institute, San Antonio, Texas, USA), EW7 (gift from Dr. Olivier Delattre, Institut Curie, Paris, France), A4573 (gift from Dr. Santiago Ramón y Cajal, Hospital Universitari Vall d'Hebron-Universitat Autònoma de Barcelona, Barcelona, Spain), TC71 and RD-ES (bought from Leibniz Institute DSMZ-German Collection of Microorganisms and Cell Cultures, Braunschweig, Germany) cell lines were cultured in RPMI 1640 (Invitrogen) supplemented with 10% heat-inactivated fetal bovine serum (Invitrogen). All cell lines were incubated at 37°C in a humidified atmosphere of 5% CO_2_ in air and checked regularly for mycoplasma infection. Exponentially growing cells within two sequential passages were used for all experiments. Cells were transfected using Lipofectamine 2000 (Invitrogen) following the protocols of the manufacturer. Transfected cells were selected with 0.4 mg/mL neomycin (Invitrogen) or 0.5 μg/mL puromycin (Sigma-Aldrich) for 14 days. CAV1 silencing was done as previously described [10]. pRS-shRNA anti-RSK1 was bought from OriGene. Antibiotic-resistant pools and individual clones were isolated for further analysis and maintained in the presence of neomycin (0.4 mg/mL) or puromycin (0.5 μg/mL). For MEK1/2 inhibitor treatment, A673 and TC71 cells were seeded and after 24 h, cells were incubated with 20 μM U0126 (CAS number 109511-58-2, LC Laboratories) in absence of serum for 24 and 48 h. Then, medium was collected for zymography and cells were harvested for protein or RNA extraction (described below).

### Western blot

ES cells were lysed with radioimmunoprecipitation assay buffer (RIPA Buffer, Thermo Scientific) containing protease inhibitors (complete, Mini; Protease Inhibitor Cocktail Tablets, Roche) and phosphatase inhibitors (PhosStop, Phosphatase Inhibitor Cocktail Tablets, Roche) and centrifuged at 13,000 *g*, at 4 °C, for 30 minutes. The protein content of the supernatants was determined with the BCA assay system (Pierce). Lysate aliquots (50 μg) were resolved by 8, 10 or 12% SDS-PAGE (depending on the size of the protein that was analyzed) and transferred onto nitrocellulose membranes (0.2 mm, Bio-Rad). After blocking with 5% skimmed milk in Dulbecco's PBS (DPBS) containing 0.1% Tween20 at room temperature for 1 h, membranes were incubated overnight at 4 °C with the appropriate primary antibody (CAV1 #610059 from BD Transduction Lab; ERK1/2 #4695, Phospho-ERK1/2 Thr202/Tyr204 #4376, MEK1/2 #4694, Phospho-MEK1/2 Ser217/221 #9154, RPS6 #2217, Phospho-RPS6 Ser235/236 #4858, RSK1 #9333, Phospho-RSK1 Thr359/Ser363 #9344, RSK2 #9340, Phospho-RSK2 Ser227 #3556 from Cell Signaling Technology, IQGAP1 #05-504 from Millipore). Blots were then incubated at room temperature for 1 h with a horseradish peroxidase-conjugated secondary antibody (goat anti-rabbit and goat anti-mouse) and the peroxidase activity was detected by enhanced chemiluminescence (Pierce) following the instructions of the manufacturer. Immunodetection of α-tubulin (#ab28439 from Abcam) was used as a loading reference. Measure of the intensity of the bands was done with Image J software (NIH).

### Gelatin zymography

Metalloproteinase activity was analyzed by gelatin zymography. In brief, cells were cultivated in serum-free medium for 48 h. Conditioned medium was then collected and concentrated to one-tenth of its original volume and electrophoresed on SDS polyacrylamide gels (10%) containing 120 μg/mL of gelatin. After electrophoresis, the gel was incubated for 60 minutes in 2.5% Triton X-100 and left overnight at 37°C in 50 mmol/L of Tris (pH 8.0)/5 mmol/L CaCl_2_. Then, the gels were stained with 0.1% Coomassie brilliant blue and destained with 10% isopropanol in 10% acetic acid, and the gelatinolytic activity was identified as transparent bands in the Coomassie brilliant blue–stained background. Measure of the intensity of the bands was done with Image J software (NIH).

### Transwell migration assay

1.5×10^5^ cells in 150 μL free-serum media were added to the top chamber of 8 μm Costar polycarbonate transwells (Transwell Permeable Supports-Corning), while in the bottom chamber 500 μL of complete media were added. After 24 h for A673 and 36 h for TC71, cells on the upper membrane surface were removed and migratory cells on the membrane underside were fixed using 70% ethanol and stained using 0.1% crystal violet solution (Invitrogen). The number of migrated cells (crystal violet stained ones) on the underside membrane was determined counting by optical microscopy. Data were presented as the average number of migratory cells in 5 high-power fields (100X). Each experiment was performed in triplicate, and then the data were averaged for statistical analysis.

### Matrigel invasion assay

8 μm Costar polycarbonate transwells (Transwell Permeable Supports-Corning) were coated with 50 μl cold Matrigel (BD Biosciences) diluted 1:20 in RMPI 1640 and placed in a 37°C incubator for 6 h. Cells were seeded, stained and counted as in the migration assay, but stopping the reaction after 60 h.

### Reverse transcription-PCR

Total RNA (2 μg), extracted using the RNeasy Mini Kit (Qiagen), was used for cDNA synthesis with SuperScript II Reverse Transcriptase (Invitrogen). Amplifications of *MMP-9* and *β-actin* were carried out using specific primers (forward primer GAGGAATACCTGTACCGCTATG, reverse primer CAAACCGAGTTGGAACCAC for *MMP-9*; forward primer CGGGACCTGACTGACTACCTC, reverse primer CTTCATTGTGCTGGGTGC for *β-actin*) designed using the Oligo 6.0 software (National Bioscience). For each set of primers, the number of cycles was adjusted so that the reaction end points fell within the exponential phase of product amplification, thus providing a semi-quantitative estimation of relative mRNA abundance. Experiments were carried out at least twice.

### Quantitative real time PCR

Quantitative reverse transcription-PCR (qRT-PCR) was performed under universal cycling conditions on an ABI 7300HT instrument (Applied Biosystems) using commercially available *MMP-9* probe (Hs00234579_m1) and mastermix (all from Life Technologies). Cycle threshold (*C*T) values were normalized to *β-actin* (ACTB). Experiments were performed at least three times and in triplicates. Relative expression level of the target gene among the different samples was calculated using the ΔΔ*C*T method [50]. Mean values and standard deviations were calculated based on the results of three biological replicates at least.

### Receptor tyrosine kinase (RTK) phosphorylation array

Total protein extracts from A673 CAV1 knockdown cells were analyzed with PathScan^®^ RTK Signaling Antibody Array Kit (Chemiluminescent Readout) (Cell Signaling Technology #7982), that allows for the simultaneous detection of 28 receptor tyrosine kinases and 11 important signaling nodes, when phosphorylated at tyrosine or other residues. Manufacturer instructions were followed. Measure of the intensity of the bands was done with Image J software (NIH).

### Sucrose gradient

Sucrose gradient was performed as described elsewhere [51]. Briefly, two 10 cm dishes of A673 and TC71 cells were washed twice with cold PBS and resuspended in 500 μl of homogenization buffer (50 mmol/L Tris-HCl, pH 7.5, 150 mmol/L sodium chloride, and 5 mmol/L EDTA), supplemented with 10 μg/mL leupeptin and 10 μg/ml aprotinin. Cells were disrupted at 4°C by nitrogen cavitation in a cell disruption bomb (Parr Instrument Company) at 800 psi for 15 minutes and collected dropwise. Afterward, cells were passed back and forth through a 22-gauge needle 25 times at 4°C. Nuclei and unbroken cells were removed by centrifugation at 1,600 *g* in a MLA-130 rotor (Beckman Coulter) for 5 minutes at 4°C. The resulting supernatant (500 μL) was mixed with an equal volume of 2.5 M sucrose and loaded at the bottom of a discontinuous sucrose gradient formed by layers of 200 μL of 30, 25, 20, 15, 10, and 5% sucrose (wt/vol) freshly prepared in homogenization buffer. Gradients were centrifuged in a TLS-55 swinging-rotor (Beckman Coulter) without brake at 166,000 *g* for 3 h at 4°C. Five fractions of 280 μl were collected from the top in addition to a final lower fraction of 700 μL by using a CentriTube Slicer (Beckman Coulter). The gradients shown in the figures are representative of at least two independent experiments.

### Immunofluorescence

For immunofluorescence, ES cells were cultured in sterile slides (Millicell EZ slide from Millipore) for 24 h until 60-70% confluence. Expression of CAV1 in ES was analyzed using a rabbit polyclonal antibody with a 1:1000 dilution (CAV1 #610059, BD). IQGAP1 expression was analyzed using an antibody at a 1:100 dilution (IQGAP1 #05-504, Millipore). Cells were fixed with 4% formaldehyde, washed thrice in Dulbecco's PBS (DPBS), permeabilized in 0.1% Triton for 2 minutes, blocked for 1 h in blocking buffer (10% fetal bovine serum in DPBS) and incubated with primary antibodies overnight. Cells were then washed thrice in DPBS for 5 minutes each followed by a 1 h incubation with secondary antibodies (Alexa Fluor 488 goat anti-mouse and Alexa Fluor 594 goat anti-rabbit; Invitrogen). Nuclei were counterstained with 4′,6′-diamidino-2-phenylindole (DAPI, Invitrogen) for 5 minutes. Then, cells were washed twice in DPBS for 10 minutes and twice in distilled water for 10 minutes, and mounted in ProLong Gold antifade reagent (Invitrogen). Photographs were taken with a Leica TCS SP5 spectral confocal microscope (argon, 405 diode and DPSS561 lasers) using a lambda blue 63×1.35 numerical aperture oil objective. Images were analyzed with the Image J software (NIH).

### Tissue samples and immunohistochemistry

Tumor samples were processed and p-RPS6 expression analyzed as described elsewhere [10]. p-RPS6 was detected with a 1:400 dilution of a phospho-RPS6 Ser235/236 antibody (#4858, Cell Signaling Technology).

### Experimental metastasis assay

The *in vivo* experimental metastasis model was performed as described previously [10]. Briefly, 2×10^6^ cells resuspended in 100 μL of saline solution were injected intravenously (i.v.) in the tail of athymic nude mice (BALB/c^*nu/nu*^) purchased from Harlan. After 45 days mice were euthanized and lungs were excised for further analysis. Tumors were fixed in 4% paraformaldehyde and embedded in paraffin. Metastases were counted under an optical microscope. Data are given as mean ±SD.

### *In vivo* orthotopic xenograft model

2×10^6^ cells resuspended in 0.1 mL of PBS were injected using a 25 gauge needle into the gastrocnemius muscles of 6 weeks old female athymic nude mice (BALB/c^*nu/nu*^) from Harlan (n=7 for each clon). The growth of primary tumors was monitored by periodical measurements of the limb using a caliper. Tumor volume was calculated according to the formula (L×l^2^/2), where L is the longer diameter and l the shorter diameter. Once primary tumor-bearing limbs reached a volume of 800 mm^3^ (18-20 days after inoculation), gastrocnemius muscles were surgically resected to reduce morbidity associated with excessive tumor growth and to allow metastases to form. Mice were maintained anesthetized in a 3% isofluorane chamber during all the surgical procedure. Tumor-bearing gastrocnemius were identified by Achilles tendon and completely resected till the knee. Afterwards, the injury was sealed using a TB10 silk suture. Mice received an i.p. injection of meloxicam (1 mg/kg) as analgesia after surgery and kept under a warm lamp until recovery that was almost complete after 15 minutes. At day 60 after inoculation mice were euthanized and lungs were harvested and fixed in 10% buffered paraformaldehyde for histopathological analysis. However, 20% of mice developed local relapses in areas adjacent to the surgery site so they were euthanized before the experiment end-point but also included in the experiment. In both metastasis models animal care procedures were followed according to the institutional guidelines for the care and use of laboratory animals. Ethics approval was provided by the locally appointed ethics committee from IDIBELL, Barcelona, Spain.

### Statistical analysis

Two-tailed Student's *t*-test was used to determine statistical significance. Unless otherwise stated, experiments were performed thrice; *p* ≤ 0.05 was regarded as significant. The analysis was done using either Microsoft Excel or GraphPad Prism software.

## SUPPLEMENTARY MATERIALS FIGURES AND TABLES





## Abbreviations

ES: Ewing sarcoma
CAV1: caveolin-1
MMP-9: matrix metalloproteinase-9
MAPK: mitogen-activated protein kinase
RPS6: ribosomal protein S6
RSK: p90 ribosomal S6 kinases
IQGAP1: IQ Motif Containing GTPase Activating Protein 1
sh: short hairpin
RTK: receptor tyrosine kinases
IF: immunofluorescence
IHC: immunohistochemistry
