# Supplementary material for: Caveolin-1 promotes Ewing sarcoma metastasis regulating MMP-9 expression through MAPK/ERK pathway
Source: Oncotarget. 2016 Jul 28;7(35):56889–903. doi: 10.18632/oncotarget.10872 (PMC5302960; doi:10.18632/oncotarget.10872)
Supplement: Supplementary file 1 [file oncotarget-07-56889-s001.pdf]

Caveolin-1 promotes Ewing sarcoma metastasis regulating MMP-9 expression through MAPK/ERK pathway

SUPPLEMENTARY FIGURES AND TABLES

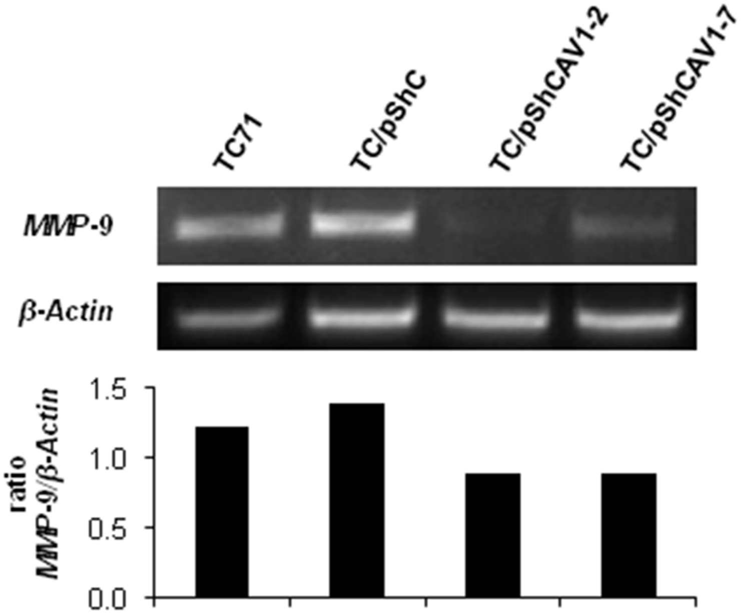

Supplementary Figure S1: *MMP-9* expression in TC71 CAV1-silenced cells. Semi-quantitative RT-PCR and corresponding quantification showing *MMP-9*/*β-Actin* ratio in the TC71 shCAV1 model.

	Stage		Tumor size		Tumor site	
	Localized	Metastatic	<8cm	>8cm	Axial	Extremity
p-RPS6 (n=26)						
Negative	8	6	4	10	9	5
Positive	10	2	5	7	7	5

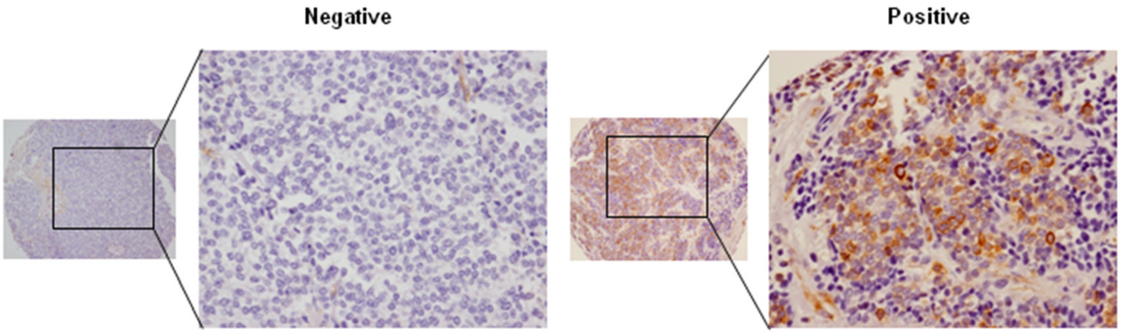

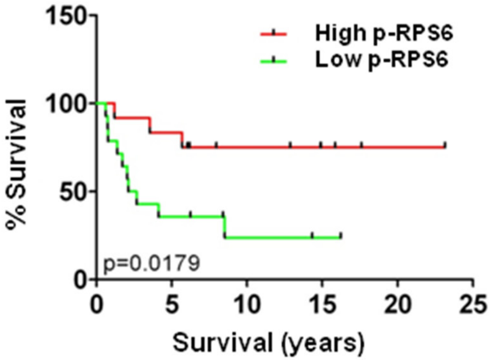

Supplementary Figure S2: High phosphorylation levels of RPS6 are a good prognostic marker in ES tumors. Above: Summary of TMA data with regard to p-RPS6 expression (negative *versus* positive) and clinical features. Middle: Example of negative and positive p-RPS6 staining of ES patient samples from the TMA (magnification, ×40; insert, ×400). Survival curve showed a positive correlation between high level of p-RPS6 and patient survival. Survival curve was generated by Kaplan-Meier method and compared by using the log rank test.

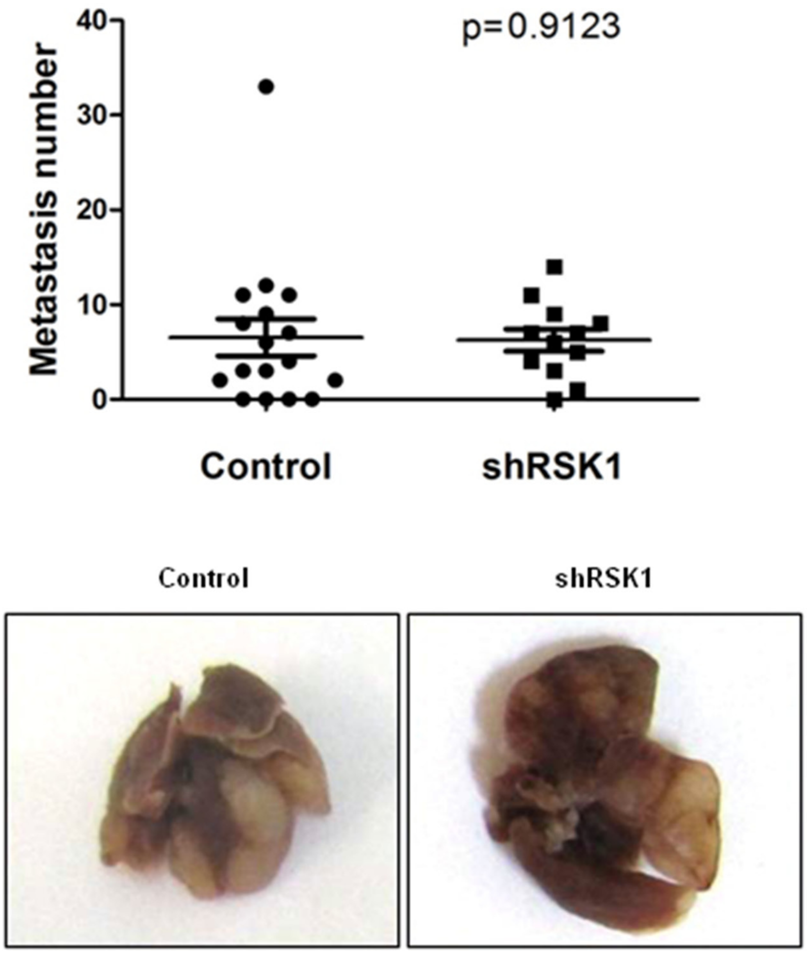

Cellular model injected	Lung metastases	Mice number
A673	8	8
ApRS 1	5	9
ApshRSK1 21	5	5
ApshRSK1 23	6	7

Supplementary Figure S3: A673 shRSK1 model did not show differences in metastasis incidence in a tail-vein injection xenograft model. Above: Number of lung metastases in mice inoculated with control (Control= A673 & ApRS1) and RSK1-knockdown cells (shRSK1= ApshRSK1-21 & ApshRSK1-23). Middle: Representative images of the lungs of each group. Below: Table showing the lung metastases and total mice numbers for every injected model.

Supplementary Table S1: Table shows the most significant receptor tyrosine kinase (RTK) up and downregulated in A673 CAV1-silenced cells

UniGene	GeneBank	Symbol	Description	Phosphorylation site detected	Gene name
Proteins inhibited in CAV1 knockdown A673 cells					
Hs.171596	BC037166	**EphA2**	Ephrin type-A receptor 2	pan-Tyr	EphA2
Hs.350321	X15262	**Ret**	Proto-oncogene tyrosine-protein kinase receptor Ret	pan-Tyr	RET, CDHF12, CDHR16, PTC, RET51
Hs.408073	M77232	**S6**	40S ribosomal protein S6	Ser 235/236	RPS6, OK/SW-cl.2
Hs.861	EU332853	**ERK1**	Mitogen-activated protein kinase 3	Thr202/Tyr204	MAPK3, ERK1, PRKM3
Hs.195659	CH471077	**Src**	Proto-oncogene tyrosine-protein kinase Src	pan-Tyr	SRC
Proteins activated in CAV1 knockdown A673 cells					
Hs.525622	AB451242	**AKT1**	RAC-alpha serine/threonine-protein kinase	Thr 308	AKT1, PKB, RAC
Hs.352548	BC126481	**IGF-IR**	IGF-like family receptor 1	pan-Tyr	IGFLR1

Supplementary Table S2: Tissue microarray patient (TMA) dataset

See Supplementary File 1
